# The Efficiency and Budgeting of Public Hospitals: Case Study of Iran

**DOI:** 10.5812/ircmj.4742

**Published:** 2013-05-05

**Authors:** Hasan Yusefzadeh, Hossein Ghaderi, Rafat Bagherzade, Mohsen Barouni

**Affiliations:** 1 School of Health Management and Information Sciences, Department of Management and Health Economics, Tehran University of Medical Sciences, Tehran, IR Iran; 2 School of Health Management and Information Sciences, Department of Foreign Lamguages, Tehran University of Medical Sciences, Tehran, IR Iran; 3 Research Center for Health Services Management, Institute for Futures Studies in Health, Kerman University of Medical Sciences, Kerman, IR Iran

**Keywords:** Efficiency, Hospital, Budgets

## Abstract

**Background:**

Hospitals are the most costly and important components of any health care system, so it is important to know their economic values, pay attention to their efficiency and consider factors affecting them.

**Objective:**

The aim of this study was to assess the technical scale and economic efficiency of hospitals in the West Azerbaijan province of Iran, for which Data Envelopment Analysis (DEA) was used to propose a model for operational budgeting.

**Materials and Methods:**

This study was a descriptive-analysis that was conducted in 2009 and had three inputs and two outputs. Deap2, 1 software was used for data analysis. Slack and radial movements and surplus of inputs were calculated for selected hospitals. Finally, a model was proposed for performance-based budgeting of hospitals and health sectors using the DEA technique.

**Results:**

The average scores of technical efficiency, pure technical efficiency (managerial efficiency) and scale efficiency of hospitals were 0.584, 0.782 and 0.771, respectively. In other words the capacity of efficiency promotion in hospitals without any increase in costs and with the same amount of inputs was about 41.5%. Only four hospitals among all hospitals had the maximum level of technical efficiency. Moreover, surplus production factors were evident in these hospitals.

**Conclusions:**

Reduction of surplus production factors through comprehensive planning based on the results of the Data Envelopment Analysis can play a major role in cost reduction of hospitals and health sectors. In hospitals with a technical efficiency score of less than one, the original and projected values of inputs were different; resulting in a surplus. Hence, these hospitals should reduce their values of inputs to achieve maximum efficiency and optimal performance. The results of this method was applied to hospitals a benchmark for making decisions about resource allocation; linking budgets to performance results; and controlling and improving hospitals performance.

## 1. Background

In their economic efforts, human beings have always focused on maximum results using minimal facilities and resources. This is called, ‘achieving a better performance’. Efficiency is a comprehensive concept whose increase has always been considered by politicians and economists to improve quality of life, welfare, peace and human prosperity. Some people believe that survival and persistence of certain political and economic systems depend on efficiency and productivity (1). Economic and social development of health sectors and the distribution of facilities are critical. Inefficiency and ineffectiveness of services, not only reduces the quality of life, but also hinders improvement and productivity in other economic sectors and results in an increase of inequality, social injustice and political dilemmas. Health sector is the most important part of the service sector and serves as an indicator of development and social welfare, thus, consideration of its economy is essential. Hospitals as the most expensive and important component of health care systems require special attention, so much that in developing countries more than 70% of health resources are allocated to hospital services (2). An increase in public expectation of economic welfare has led to an increase in demand for health services. Therefore, regarding scarce resources and facilities, it is crucial to make the best use of the existing facilities to reduce the gap between supply and demand. Efficiency is the most important and common mechanism for evaluating and measuring the performance of enterprises such as hospitals, so in the past few decades, researchers in different fields of social sciences, particularly economics and management have focused on the performance of different economic sectors, firms and economic entities at micro levels through measuring and estimating their efficiency (3). Productivity and efficiency are important resources for economic development; therefore, they must be reviewed and analyzed in the health sector. Calculation of technical efficiency and recognition of factors affecting hospital efficiency are complementary measures for quality and quantity improvements. Elimination of factors involved in hospital inefficiency can, without adding agents, increase efficiency, enhance service delivery and help hospital administrators make more realistic, efficient and better decisions (4). The Data Envelopment Analysis (DEA) method can be an appropriate model for the operational budgeting of governmental departments, such as schools, banks, hospitals and so on, for which information on prices rarely exists or are incomplete (5). Operational budgeting process (performance based budgeting) is estimated and calculated based on operational classification of organizations' current costs and in terms of functions and activities in form of workload of each organizational unit and the measurement of the costs of each activity for efficient production of goods or services. The most important feature of operational budgeting is that it shows the relationship between the allocated funds of each program and the results of its implementation. Moreover, operational budgeting adds saving and effectiveness factors to aspects of traditional budgeting. This type of budgeting identifies and lists all direct or indirect activities in any program and offers accurate estimation of the costs of each activity. It also seeks to link performance indicators to resource allocation based on achieving obvious and measurable results. Establishing logical and technical links between performance indicators and resource allocation is necessary in this method of budgeting (6). Calculating efficiency and quantifying performance allows managers to oversee the trend of changes, identify potential problems and take timely corrective actions. Hospital systems as one of the most important and influential sectors in the society have a critical role in health promotion and because of the increased demands and limited resources it is necessary to accurately calculate efficiency and productivity. Studies conducted by Gannon in Ireland (2005), Hofler and Folland in the United States (1995), Lina in Finland (1997), Parkin and Hollingsworth in Scotland (1995), Goodarzi (2007), Saber Mahani (2009), Zohreh Kazemi (2009) and Mohsen Barouni (2012) in Iran, all emphasized the use of the DEA method for assessing hospital efficiency. In this study the DEA technique was implemented to measure efficiency and estimate optimal use of resources in hospitals (7, 8).

## 2. Objective

The aim of this study was to assess the technical scale and economic efficiency of hospitals in the West Azerbaijan province of Iran and Data Envelopment Analysis (DEA) was used to propose a model for operational budgeting.

## 3. Materials and Methods

**Figure 1.**
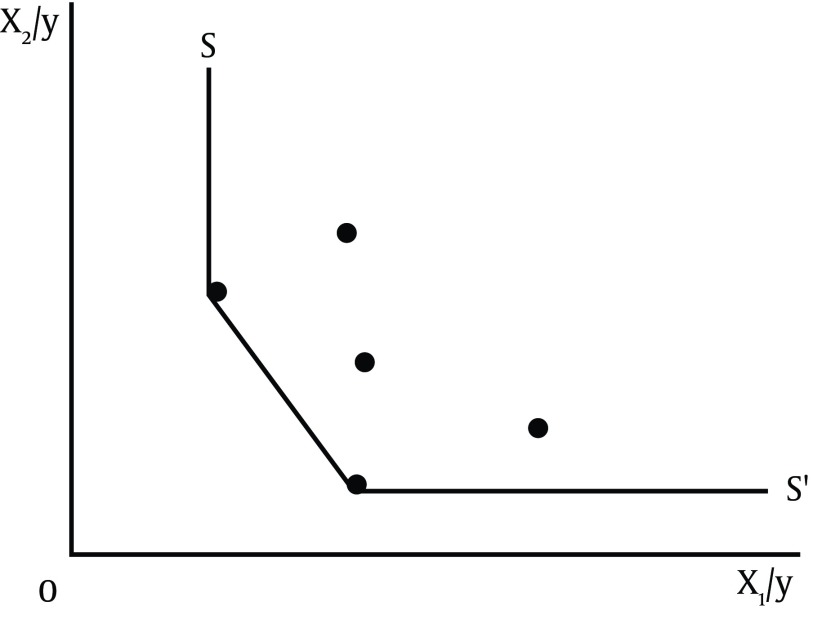
Same Production Function of Farrell

In their paper, Charnes, Cooper and Rhodes, operation research specialists, (1978), measured productivity and efficiency via Data Envelopment Analysis (DEA) which was based on a series of optimization and a linear programming known as the non-parametric method. In this method, an efficient frontier curve is made of a series of points determined by linear programming (9). Farrell, for the first time, showed how to get the same production function through geometry. He stated that if each point in Figure 1 represents the use of production factors X1 and X2 to produce the output Y in different enterprises, the connection of the points closer to the origin and axes creates a convex function with no points under it; this curve is called efficient isoquant production function. This coating surface encompasses Pareto optimum points (Pareto Efficiency) and a series of efficient units in production. If the production of an output Y requires more than two production factors X1 and X2, the geometrical drawing of isoquant production function curve would be very difficult; indeed DEA was produced to overcome such problems (10).

In cases where firms require more than two production factors to produce outputs, each decision making unit is considered as a point in space. The dimension of each point are determined by the number of production factors and its coordinates are specified by production factor level. Furthermore, using a linear programming, a decision making unit is selected as an investigation unit which is compared with other decision making units (other space points). Therefore, it is possible to assess the efficiency of points off or on this curve, which are called set of efficient points (11). To determine the points, two assumptions of fixed and variable returns to scale are used to either maximize the objective functions (output), considering certain inputs, or minimize the inputs using its duality, that is the given outputs. The linear programming method, after a series of optimization, specifies whether the desired decision making unit is located on the efficiency line or otherwise. Thereby, efficient and inefficient units are distinguished. DEA in the isoquant production function estimation does not require a particular default shape. This method is used to compare the efficiency of a firm relative to others (12). In this study, the efficiency of selected hospitals was estimated through a non-parametric approach of input orientated of DEA and variable returns to scale assumption. Linear programming is defined by:

Minλ,OS,IS (M1′ ∙ OS+K1′ ∙ IS)

St:-yt+Yλ-OS = 0‚

Θxt-Xλ-OS = 0

N1′∙λ ≤ 0, λ ≥ 0, OS ≥ 0 ‚ IS ≥ 0

In the above equation, the first constraint shows that the product surplus for each firm would be zero, if -yt + Yλ equals zero. The second limitation indicates that the production factors surplus will be zero, if the term Θxt - Xλ is zero. The third constraint expresses variable returns to scale. λis a ×N1 vector of fixed numbers indicate weights of the reference set. IS and OS refer to input and output slacks. DEA model with variable returns to scale (VRS) assumption can distinguish between scale efficiency and pure efficiency. In other words, technical efficiency can be analyzed from pure efficiency and scale efficiency through solving linear programming models with two assumptions of constant and variable returns to scale, that is:

Value of technical efficiency (with CRS assumption) = amount of technical efficiency (with VRS assumption) × scale efficiency

Studies have shown that operational budgeting system is a changing and evolving concept that cannot be examined in simplified terms and non-dynamic relationships. Budgeting process is an activity affected by political options and numerous environmental variables, that is, the allocation of limited resources to meet needs and priorities. Performance information can only be one of the factors that make up infrastructure decisions. Therefore, hospitals budget is divided into inevitable and efficiency budget (13). The inevitable budget of hospitals is determined according to indicators such as the number of official staff, active beds, regional balanced indices (as area deprivation index), historical trends and so on; therefore, it is necessary to distribute a part of the overall budget of health sectors to hospitals based on efficiency criteria. Obviously, the share of each case of inevitable and efficiency budget can vary in line with the policy of the executive institute and the suggestions made by managers. If the total distributable budget among hospitals is shown with I, the inevitable budget with A and the efficiency budget of institute with B; and if w and v indicate their weight coefficients; then, the distributing budget is equal to:

I = Av + BW

Where w and v are arbitrary coefficients and are annually determined in accordance with management suggestion and current situation and different values between zero and one can be selected. Thus:

V+W = 1

This was a descriptive-analytical (cross-sectional and retrospective) study conducted in 2009. The research population consisted of 23 hospitals affiliated to Urmia University of Medical Sciences including Imam Khomeini, Motahhari, Taleghani and Psychiatry of Urmia; Khatam of Salmas; Qamar bany Hashem; Madani and Ghra zyaaldyn of Khoy; Beheshti of Chaldoran; Fajr, Qods of Maku; shohada of Showt; Imam Khomeini of Poldasht; Imam Khomeini of Naqadeh; Imam Khomeini of Mahabad; Abbasi and Hazrat Fatima of Miandoab; Rasy of Shahyndez; Shohada of Takab; Qolypor of Bukan; Imam Khomeini of Piranshahr and Nabi akram of Oshnavieh. Input variables included the number of active beds, doctors and other personnel and output variables encompassed out patients admission and occupied day beds in studied hospitals. The data were collected using available documents in hospitals and were analyzed using DEA and Deap2,1 software. In this study, both slack and radial movements of inputs were estimated; and in addition to determining the efficiency of the selected hospitals, the surplus or excessive use of inputs was calculated as well. Finally, a new model was designed with variable returns to scale (VRS) assumption for operational budgeting of hospitals and health sectors. In order to observe ethical considerations, the results are shown with relevant numbers and if necessary, the information for each hospital will be presented to their managers.

## 4. Results

In this study, the DEA model (based on the minimization method of production factors and with the assumption of variable returns to scale (VRS)) was used with two outputs and three inputs. Deap2,1 software results are given in the following tables. According to Table 1, it is possible to evaluate hospital performance based on the technical efficiency index and compare this between hospitals. The average technical efficiency score of the hospitals calculated with DEA method was 0.584, which indicated that some hospitals did not work effectively and their capacity for efficiency promotion without any increase in costs and with the same amount of inputs was about 41.5 %.

**Table 1.** Rating of Studied Hospitals Based On Technical Efficiency Using the DEA Model and VRS

Hospital	Efficiency			Returns to Scale
	Technological	Managerial	Scale	
**9**	1	1	1	CRS
**19**	1	1	1	CRS
**21**	1	1	1	CRS
**22**	1	1	1	CRS
**4**	0.944	1	0.944	IRS
**20**	0.871	0.997	0.874	IRS
**18**	0.733	0.768	0.955	DRS
**7**	0.618	1	0.618	DRS
**14**	0.612	1	0.612	DRS
**15**	0.585	0.879	0.666	DRS
**23**	0.582	0.597	0.975	IRS
**8**	0.576	0.588	0.98	DRS
**13**	0.505	1	0.505	DRS
**2**	0.413	1	0.413	DRS
**16**	0.413	0.559	0.739	DRS
**10**	0.398	0.542	0.734	DRS
**17**	0.368	0.386	0.954	IRS
**12**	0.36	0.742	0.486	DRS
**6**	0.359	1	0.359	DRS
**1**	0.3	1	0.3	DRS
**5**	0.299	0.357	0.837	DRS
**3**	0.269	0.315	0.853	DRS
**11**	0.238	0.256	0.932	DRS
**Mean**	0.584	0.782	0.771	

Hospitals 9, 19, 21 and 22 were the most efficient hospitals (with index 1) while hospital 11 was the least efficient one (0.238); in fact 17.3 % of hospitals were fully efficient. Also in this part of the research, return to scale was measured which shows the rate of increase in production provided that all other resources are equally increased. It also revealed three cases: 1) constant return to scale (CRS) in 17.3 % of hospitals, where equal increase in all production factors led to the same amount of increase in production, 2) increasing returns to scale (IRS) in 17.3 % of hospitals, where equal increase in all production factors resulted in more production and 3) Decreasing returns to scale (DRS) in 65.2 % of hospitals, where equal increase in all production factors led to less production. For inefficient hospitals, DEA method identified some production factors and products which indicated a decrease in the use of production factors or an increase in the amount of products (minimization is used in health sectors). According to the results obtained from the Deap2,1 software, most surplus of doctor and bed inputs were related to hospital 11 and the highest rate surplus of other staff was related to hospital 3. Tables 2 and 3 show the weight rate of all reference hospitals (hospital peers) for non-efficient hospitals and indicate that the use of production factors in each reference hospital is less than a non-efficient hospital. For example, reference hospitals for a non-efficient hospital (8) are hospitals (14) (9) whose weights are 0.631 and 0.369, respectively. The optimal values of inputs are determined with the application of these coefficients and hospital (8) can achieve maximum efficiency. Moreover, information related to reference hospitals can be used for a better assessment of inefficient hospitals.

**Table 2.** Reference Hospitals (hospital peers)

Hospital	Efficiency			Returns to Scale
	Technological	Managerial	Scale	
**1**	1			
**2**	2			
**3**	7	9	19	
**4**	9	21		
**5**	9	13	22	
**6**	7	2	13	1
**7**	7			
**8**	19	9		
**9**	9			
**10**	7	14	13	9
**11**	7	9		
**12**	14	13	9	
**13**	13			
**14**	14			
**15**	13	14	9	
**16**	7	9		
**17**	21	9	22	
**18**	19	9		
**19**	19			
**20**	21	9	22	
**21**	21			
**22**	22			
**23**	21	9	22	

**Table 3.** Hospital peer weights

Hospital	Efficiency			Returns to Scale
	Technological	Managerial	Scale	
**1**	1			
**2**	1			
**3**	0.203	0.599	0.198	
**4**	0.939	0.061		
**5**	0.627	0.159	0.215	
**6**	0.226	0.103	0.46	0.211
**7**	1			
**8**	0.369	0.631		
**9**	1			
**10**	0.327	0.072	0.023	0.579
**11**	0.078	0.922		
**12**	0.694	0.158	0.148	
**13**	1			
**14**	1			
**15**	0.067	0.123	0.811	
**16**	0.458	0.542		
**17**	0.234	0.565	0.201	
**18**	0.26	0.74		
**19**	1			
**20**	0.301	0.177	0.522	
**21**	1			
**22**	1			
**23**	0.72	0.148	0.132	

In this study, Koopmans definition was used for calculating efficiency. In other words, slack and radial movements were both estimated and finally surplus or excessive use of inputs was calculated. The results are summarized in Table 4.

**Table 4.** Average Amount of Over the Need Utilization to Separate Input Using the DEA-VRS Model

Input/average	Physician	Other personnel	Active Bed
**Original value**	26	115	116
**Projected value**	17	86	85
**Slack movement**	9	29	31

According to the results, the most surplus of input was related to bed input and the lowest to physician input. As was expressed distributing budget is equal to: I = Av + BW, where BW shows efficiency budget of an institute. If C is all allocated funds to hospitals based on efficiency criteria and C = BW, hospital I will take the CI currency based on the proposed allocated pattern; that is the sum of allocated budget to independent sectors which is equal to the total budget allocated to state institutes or universities:

C =∑^n=23^ᵢ_=1_ c_i_

The following steps should be taken to determine each hospital's share of efficiency budget: if the sum of technical efficiency is E and EI represents technical efficiency index of hospital I:

E = ∑^n=23^ᵢ_=1_ e_i _ (I = 1,………,11)

CI share of each hospital of efficiency budget is equal to:

C_i_ = C × e_i_/E

## 5. Discussion

It was found that the evaluated hospitals did not work efficiently and the capacity for efficiency promotion without any increase in costs and with the same amount of inputs was about 41.5 %. Thus, the hospitals had surplus capacity. Most excess use of resources or additional inputs was related to active bed input. The lowest technical efficiency was related to hospital 11 with a technical efficiency of 0.238 and a decreased return to scale. The average technical efficiency score of the hospitals was 0.584. In other words, these hospitals can provide the same current level of outputs using 58.4 % of their resources. The mean score for pure technical efficiency (managerial efficiency) of hospitals was 0.782, that is without increasing inputs and only with good and wise management and the effort of employees; the efficiency can be increased up to 21.8%. The average scale efficiency score for hospitals was 0.771, so hospitals should act efficiently to have increasing return to scale and increase their services, because with the assumption of constant factors for production, output will exceed inputs. Therefore, long-term marginal cost and thereinafter long-term total cost will decrease and there will be an economic justification for the increased services. In studies conducted by Goodarzi et al. in hospitals affiliated to the Tehran University of Medical Sciences, Saber Mahani in Kerman University of Medical Sciences hospitals and Zohreh Kazemi in hospitals of South Khorasan province, the calculated mean scores for technical efficiency were 0.972, 0.912 and 0.886, respectively, which were more than those of Urmia hospitals. Barouni et al. performed the Provincial human development index, a guide for resource allocation using the DEA method. The results showed the national mean for the HDI in 2001 was 0.717 while it increased to 0.747 in 2009, showing an improvement of 4.2%. Except for one province, all others had an improved human development index; although the level of improvement was very small in some provinces. Low ranked provinces, such as Sistan & Baluchistan and Kurdistan remained at the bottom in 2009. However some provinces such as Bushehr with developing oil industries, or those purposively benefited from national oil income showed good growth. In some provinces, such as Hormozgan, out-migration of manpower to its neighboring province, Bushehr, was associated with a decrease of the provincial income level. The number of efficient provinces increased from 5 to 13 (43% of all provinces) in 2009. According to these findings, in hospitals with maximum technical efficiency (with index 1), the original and projected values of inputs were the same and input surplus was zero. On the other hand, in hospitals with a technical efficiency less than one, the original and projected values of inputs were different and they had surplus of input. Therefore, they should reduce their surplus from original values to reach optimal performance. Hospitals with an efficiency less than one, for example, hospital 8, had surplus in physician, other personnel and active bed inputs, and should reduce 72.8 % of their original values of doctor input, 45.9 % of other personnel input and 41.2 % of active bed input. In fact, they should reduce doctor input from 25 to 6, other staff from 71 to 38 and active bed from 70 to 41 and eliminate 19 doctors, 33 other personnel and 29 active beds that have no roles in production. Since the output (number of patients) is not controlled by hospitals, it is not quite practical to use output maximization, but it is possible to find information on optimized output and take measures in competing with other hospitals to increase service quality, enhance customer satisfaction, attract patients and improve service volume. Considering the surplus capacity of production factors in hospitals, it seems that reduction of these factors should be done through comprehensive planning, taking all aspects into consideration. More than half of the health staff work in hospitals which consume a major part of the fixed costs of the health sector. Therefore, proper planning on how to use the resources and remove surplus manpower based on the DEA will have a significant role in reducing costs of hospitals and health sectors (while in some hospitals, despite the surplus capacity, new staff are still recruited). One of the major limitations of this study was the exclusion of the severity of diseases and quality of care provided to patients because there were no data related to cases across hospitals in the country. As a result, cases that had significant influence on hospitals performance were not included in the study. Hence, the studied indices could not determine the complexity of activities or show hospital performance in real terms; for example, some hospitals may treat some easy and daily cases and refer complicated ones to other hospitals (15). Therefore, it is recommended to conduct studies to achieve and define case mix indices (the combination of different patients treated in a particular hospital) in Iran. Operational budgeting system is a system for producing and exchanging current and future functional information (real and expected results); on the other hand it is a system of purchasing the expected results with government funds. The DEA technique can be used as a framework for the inclusion of performance indicators in the process of operational budgeting and can be implemented in hospitals. Performance indicators should include comparative criteria to determine hospitals status with reference to their competitors, partners and what is considered by experts. Comparison is very effective in determining goals and performance motivation and recognizing organizational excellences, and thus it is the only way to determine the adequacy of development (16). The DEA technique can be used to link budget to performance and compare each hospital's performance with the others. It is also possible to assess the existing and former status of hospitals. Considering the deficiencies of this model and the lack of statistical tests to confirm the findings, it is suggested to use the results of parametric approaches in modeling non-parametric models, to consider inputs, which are not located in the third area of production and are meaningful in parametric methods.
